# The Expression of NOX4 in Smooth Muscles of Small Airway Correlates with the Disease Severity of COPD

**DOI:** 10.1155/2016/2891810

**Published:** 2016-08-30

**Authors:** Xianyan Liu, Binwei Hao, Ailing Ma, Jinxi He, Xiaoming Liu, Juan Chen

**Affiliations:** ^1^Department of Pulmonary and Critical Care Medicine, The General Hospital of Ningxia Medical University, Yinchuan 750004, China; ^2^Ningxia Medical University, Yinchuan 750004, China; ^3^Binzhou People's Hospital, Binzhou, Shandong 256600, China; ^4^Department of Pathology, The General Hospital of Ningxia Medical University, Yinchuan 750004, China; ^5^Department of Thoracic Surgery, The General Hospital of Ningxia Medical University, Yinchuan 750004, China; ^6^Institute of Human Stem Cell Research at the General Hospital of Ningxia Medical University, Yinchuan, Ningxia 750004, China

## Abstract

Airway smooth muscle (ASM) remodeling is a hallmark in chronic obstructive pulmonary disease (COPD), and nicotinamide-adenine dinucleotide phosphate (NADPH) oxidases (NOXs) produced reactive oxygen species (ROS) play a crucial role in COPD pathogenesis. In the present study, the expression of NOX4 and its correlation with the ASM hypertrophy/hyperplasia, clinical pulmonary functions, and the expression of transforming growth factor *β* (TGF-*β*) in the ASM of COPD small airways were investigated by semiquantitative morphological and/or immunohistochemistry staining methods. The results showed that an elevated expression of NOX4 and TGF-*β*, along with an increased volume of ASM mass, was found in the ASM of small airways in COPD patients. The abundance of NOX4 protein in the ASM was increased with disease severity and inversely correlated with the pulmonary functions in COPD patients. In addition, the expression of NOX4 and ASM marker *α*-SMA was colocalized, and the increased NOX4 expression was found to accompany an upregulated expression of TGF-*β* in the ASM of small airways of COPD lung. These results indicate that NOX4 may be a key regulator in ASM remodeling of small airway, in part through a mechanism interacting with TGF-*β* signaling in the pathogenesis of COPD, which warrants further investigation.

## 1. Introduction

Chronic obstructive pulmonary disease (COPD) is one of the most common diseases characterized by persistent airflow limitation, along with the presence of both oxidative stress and airway inflammation [1, 2]. The prevalence of COPD is remarkable variation from 7.8% to 19.7% among persons over age 40 [1, 2]. In China, the overall prevalence of COPD was 8.2% as defined by the criteria of Global Initiative for Obstructive Lung Disease [3–5]. Although substantial advances in the pathophysiology, diagnostics, and treatment of COPD have been made, and environmental exposure and primarily cigarette smoking have been implicated as major risk factors for the pathogenesis of COPD [1, 2], mechanisms underpinning the pathogenesis of this disease remain far from being understood. To date, interactions of multiple genetic and environmental factors have been recognized to contribute to the development and progression of this partially reversible lung disorder. Mechanisms involving chronic airway inflammation, oxidant and antioxidant imbalance caused by overwhelming oxidative stress, protease and antiprotease imbalance, cell apoptosis, and airway remodeling have recently been suggested as main drivers in the pathogenesis of COPD [6]. Among these pathogenic insults, the oxidative stress with an imbalance between oxidants and antioxidants has gained an increasing attention, which may provoke pathological reactions causing COPD [6].

The oxidative stress is referred to as elevated intracellular level of reactive oxygen species (ROS) that causes damage to cell organelles, which has been linked to signaling in both of pathologies of diseases and the maintenance of physiological functions [6]. It has been demonstrated that ROS is a class of intracellular signaling molecules that play important roles in cell growth, differentiation, apoptosis, and gene expression, as well as the activation of cell signaling cascades [6]. However, excessive ROS accumulation can also induce oxidative stress and result in cell damage [6]. In terms of ROS production, several enzymes have been identified to be involved in evoked levels of ROS implicated in pulmonary disorders; among these ROS-producing enzymes, the members of nicotinamide adenine dinucleotide phosphate (NADPH) oxidases (NOXs) are the most investigated ones, which play crucial roles in the maintenance of lung integrity [7]. Mounting evidence has revealed that an inappropriate expression or activation of NOXs was implicated in many types of pulmonary diseases including acute lung injury, idiopathic pulmonary fibrosis (IPF), asthma, and COPD [2, 6, 7].

NOXs are a class of membrane protein family that constitutes seven members including NOX1-NOX5, dual oxidase (DUOX) 1/2 (DUOX1/2), which are a primary source of ROS within the lung and cardiovascular system where NOXs are implicated in many cellular functions ranging from the regulation of signaling transduction and gene expression [8]. Of note, these NOX proteins have a cell type-dependent subcellular localizations and distributions [9, 10]. Differs from other NOX family members, NOX4 has unique enzymatic properties, which is constitutively activated and does not require the assembly of an active enzymatic complex by recruitment of cytoplasmic regulator proteins that are critical for the activation of other NOX family members [11, 12]. The subcellular localization, tissue distribution, and impact on ROS signaling of NOX4 are also distinct from other NOX enzymes [9, 11, 12]. An increasing number of investigations have revealed that NOX4 is involved in various cellular functions including oxygen sensing, cell proliferation and differentiation, apoptosis, fibrosis, and inflammation [13–17]. An excessive expression of NOX4 was reported in both of cardiovascular and pulmonary diseases, including atherosclerosis, pulmonary fibrosis, pulmonary hypertension, and COPD [16, 18–20]. In addition, NOX4 expressed in mesenchymal cells, epithelial cells, and smooth muscle cells of lower airways was required for maintenance of the differentiated vascular smooth muscle cell phenotype. A dysregulated NOX4 expression was reported to contribute hypertensive vascular remodeling and airway smooth muscle (ASM) hypercontractility in asthmatic lungs, in which an ASM hypertrophy was a hallmark of airway remodeling of asthmatic pathogenesis [13, 21–23].

Airway remodeling has been attributed to the proliferation of airway epithelial cells, proliferation and hypertrophy of ASM cells, deposition of extracellular matrix (ECM), and differentiation of fibroblasts into muscle fibroblasts, which ultimately results in airway fibrosis and obstruction. In this context, the proliferation, hypertrophy, and functional accentuation of ASM cells play a key role in the procession of airway remodeling [24, 25]. Recently, several lines of evidence demonstrated that an expression of NOX4 was significantly elevated in IPF-derived human lung fibroblasts treated with TGF-*β*1 [26] and ASM in asthmatic patients [27]. The activation of NOX4 was along with the generation of hydrogen peroxide (H_2_O_2_), myofibroblast differentiation, contractility, and ECM production in response to TGF-*β*1 in human myofibroblasts, and therapeutic targeting NOX4 could protect animal models from injury-provoked pulmonary fibrosis [26]. Consistently, genome-wide microarray analysis also evidenced more abundant NOX4 transcripts in the primary ASM of human asthmatic lung relative to healthy controls [27]. These studies clearly indicate that NOX4 may play critical roles in the pathogenesis of pulmonary arterial remodeling and pulmonary hypertension, myofibrosis in IPF, and ASM hypercontractility in asthma. Given the fact that the ASM cell layer is thickened in asthma, it may imply that NOX4 also plays a role in ASM hyperplasia and hypertrophy in the lungs of other chronic pulmonary diseases.

Small airway remodeling has been acknowledged as the core hallmark of COPD. An increased mucous metaplasia and submucosal gland hypertrophy, peribronchial fibrosis, and an increased ASM mass were accompanied by the disease severity. Intriguingly, a most recent study revealed that the large airway remodeling was also involved in the pathogenesis of COPD, which was supported by observations of a greater deposition of ECM proteins in the subepithelial layer and a larger smooth muscle area in human COPD lungs, in comparison with control smoker subjects [28]. However, this finding was different from a previous study in which no difference in the smooth muscle area was found in the cartilaginous airway between COPD patients and the control group [29]. On the other hand, an increased ASM mass was found in large airways of severe asthmatics and in small airways of patients with COPD [30]. These controversial results indicate that the role and mechanism of ASM remodeling in COPD are less well understood and need to be further investigated, despite the fact that the ASM hyperplasia and hypertrophy are thought to contribute to the airflow limitation in COPD.

Given functions of NOX4 in ROS production and pathogenesis of pulmonary disorders, as well as ASM hyperplasia and hypertrophy in asthmatic airway remodeling, we therefore hypothesized that the NOX4 may also have an implication in ASM remodeling of small airway of COPD lung. To test it, we explored the expression of NOX4 in ASM of small airways from COPD patients in the present study. Our results demonstrated an elevated NOX4 in the ASM of small airways in COPD lungs, in which NOX4 was strongly correlated with the degree of small airway remodeling in COPD lungs.

## 2. Materials and Methods

### 2.1. Ethics Statement

Human samples were collected with a protocol approved by the Ethic Committee for the Conduct of Human Research at Ningxia Medical University (NXMU-H-201309). Written consent was obtained from every individual according to the Ethics Committee for the Conduct of Human Research protocol. All participants provided a written informed consent for the publication of the data. The Ethics Committee the Conduct of Human Research at Ningxia Medical University approved the consent procedure for this study (NXMU-H-201309).

### 2.2. Subjects

Basic demographic information was collected using a specifically designed questionnaire after obtaining written informed consent. Smoking status was defined as nonsmokers (never smokers), former smoking (quit smoking since at least 6 months), and current smoking (smoking at least one cigarette daily for more than 6 months). The cigarette consumption was calculated by taking the number of packs of cigarettes smoked per day and multiplying it by years of smoking (pack-years). Consecutive patients who were about to undergo lung volume reduction surgery (LVRS), pneumonectomy, or lobectomy for suspected primary lung cancer on the surgical waiting list were recruited in the General Hospital of Ningxia Medical University, Yinchuan, China, from January 2010 to December 2013. Furthermore, the information regarding the surgical procedure was also collected. A portion of grossly normal lung tissue with a size of approximately 1.0 cm^2^ in area and 0.5 cm of thickness was collected from the distal end of lesion (≥5.0 cm) during the operation process. The sample was immediately placed in ice for preservation and the rest of the surgical piece followed a regular protocol. Standard pulmonary function testing was performed on all subjects before surgery performance. The pulmonary function was ascertained by measuring the postbronchodilator forced vital capacity (FVC) and forced expiratory volume of one second (FEV1), using a MasterScreen PFT spirometer system (CareFusion, San Diego, CA, USA) essentially according to ATS guidelines [31]. COPD and the disease severity were defined according to the criteria of Global initiative for Obstructive Lung Disease (GOLD) [1, 16]. All asthmatic subjects or other allergic disorders were excluded from the analysis in this study. The Ethics Committee the Conduct of Human Research at Ningxia Medical University approved the consent procedure for the recruitment. The demographics of subjects were listed in Table 1.

### 2.3. Histochemistry and Immunohistochemistry Analysis

Lung tissues were processed for paraffin embedding and/or embedded in optimal cutting temperature compound (OCT). Transverse airway sections were from paraffin-embedded and/or OCT-embedded lung tissues and were cut at a thickness of 4 *μ*m for hematoxylin and eosin (HE) histochemistry (includes immunohistochemistry, IHC) and immunofluorescent (IF) staining, respectively. Small airways were defined as membranous bronchioles, without cartilage or glands, and with an internal diameter less than 1.0 mm. 10 to 30 small airways per subject were evaluated in this study. The stained slides were analyzed using the Olympus light microscope BX51 (Olympus China, Beijing, China) and images were examined using Image-Pro Plus 6.0 (IPP6.0) software (Media Cybernetics, Silver Spring, MD, USA). Parameters of the thickness of ASM (*T*)/external diameter of small airways (*D*) were measured, and the percentages of *T*/*D* (WT%) and area of ASM/transverse area of small airway (WA%) were calculated using 5 randomly selected high magnification (400x) fields of each section as previously described [32, 33]. The number of ASM cells was counted by Image-J using cell counter mode [34]. Computer-assisted quantification of the staining in the selected areas was performed in images with a final magnification of 400.

Tissue sections from all subjects were stained with HE for histopathologic examination. Immunohistochemistry was performed on paraffin-embedded tissues as described elsewhere [35]. Briefly, the paraffin-embedded sections were deparaffinized and rehydrated through graded alcohol solutions. Tissue sections were microwaved in 10 mM sodium citrate pH 6.0 for 15 minutes and cooled down to room temperature (RT) for antigen retrieval. Followed by treating sections with 0.3% hydrogen peroxide in phosphate buffered saline (PBS) for 20 minutes to inactivate endogenous peroxidase before they were blocked with blocking buffer (5% donkey serum in PBS) for additional 2 h at RT. The primary antibody was then applied (diluted in blocking buffer) on the section and incubated overnight at 4°C. Paralleled sections incubated with normal rabbit IgG were used for negative controls. After washing for 3 × 5 min in PBS, sections were incubated with peroxidase labeled appropriate secondary antibodies (ZSGB-Bio, Beijing, China) (1 : 200 in blocking buffer) for 30 minutes at RT. The signal of interest was developed with 3,3′-diaminobenzidine (DAB) peroxidase substrate, followed by counterstaining with hematoxylin if it was applicable. The stained sections were examined and photographed. Five randomly selected fields of each slide at magnification of 200 were used for analyzing the positive staining [32]. The obtained images were then for a semiquantitative analysis of the expression of protein of interest by measuring the integrated absorbance (IA) or optical density (OD) using the IPP6.0 software, and the average optical density (AOD) values of each sample were used as an index of the expression of proteins [32, 36]. The primary antibodies used in IHC included rabbit anti-*α*-SMA (1 : 200, Abcam, Cambridge, MA, USA), mouse anti-TGF-*β*1 (1 : 200, Abcam), and rabbit anti-NOX4 (1 : 200, Novus Biotech, Littleton, CO, USA). Other rabbit primary antibodies to PCNA, collagen I, collagen IV, laminin, and fibronectin were products of ZSGB-Bio LTD (1 : 50, Beijing, China).

### 2.4. Dual Immunofluorescence

To identify the colocalization of NOX4 and *α*-SMA expression in ASM, double immunofluorescent staining was performed on frozen tissue sections. The sections were permeabilized with 0.2% Triton X-100 for 20 minutes at RT, followed by applying block buffer (5% donkey serum in PBS) to block nonspecific antibody binding for 1 hour at RT. Primary antibodies to mouse anti-NOX4 (1 : 100, ZSGB-Bio LTD, Beijing, China) and rabbit anti-*α*-SMA (1 : 200, Abcam, Cambridge, MA, USA) were coincubated overnight at 4°C in blocking buffer. Primary antibody binding was detected using the appropriate FITC or Texas Red-labeled secondary antibody (Jackson ImmunoResearch Laboratories, West Grove, PA, USA). Section specimens were then mounted in Vectashield Mounting Medium with DAPI to demarcate the nucleus (H-1200, Vector Laboratories, Burlingame, CA). Images were acquired using a digital camera attached to a fluorescence microscope.

### 2.5. Western Blotting Analysis

One mL of ice-cold RIPA buffer (50 mM Hepes, pH 7.6; 1 mM EDTA; 0.7% deoxycholate; 1% NP-40; 0.5 M LiCl) containing a protease inhibitor cocktail (Roche, Basel, Switzerland) was added to approximately 300 mg of lung tissue. Samples were then homogenized by sonication and rotated at 4°C for 2 hours, followed by centrifugation at 10,000 g for 10 min at 4°C. Supernatants were harvested and protein concentrations were determined using a BCA protein assay kit (Pierce, Rockford, IL, USA). The tissue lysates (75 *μ*g) were resolved by a 10% sodium dodecyl sulfate- (SDS-) polyacrylamide gel (SDS-PAGE), followed by being transferred to a PVDF membrane (Millipore, USA). The membranes were probed with antibodies to respective proteins of interest. The blots were developed using the enhanced chemiluminescence (ECL) reagent (Amersham Biosciences, Piscataway, NJ, USA) after they were incubated with the appropriate peroxidase labeled secondary antibodies. Immunoreactive signal was acquired by BioSpectrum AC image system (BioRad China, Shanghai, China).

### 2.6. Statistical Analysis

SPSS17.0 analysis software (SPSS, Chicago, IL, USA) and/or the GraphPad Prism version 5 software (Version 5.0, GraphPad Software Inc., La Jolla, CA, USA) were used for the statistic analysis. All data were expressed as mean ± standard deviation (±SD). The data were analyzed with normality test first, followed by variance homogeneity test. Two independent samples *t*-test was applied for homogeneity of variance, and Welch's approximate *t*-test was conducted for an unequal variance. For comparison of multiple means, one-way ANOVA was employed among groups, and LSD-test was performed between two groups for homogeneity of variance; and Hotelling's T2 test was used for an unequal variance. The Spearman correlation analysis was used for correlated analysis between two groups. Fisher's exact test was employed for comparison of categorical data (*n* < 40). A *p* < 0.05 was considered statistically significant.

## 3. Results

### 3.1. Demographics Data

36 patients were recruited in COPD group according to presurgical spirometry results, including 6 severe COPD patients who underwent LVRS and 30 COPD patients who also were suspected to have lung cancer and underwent curative surgical resection. The distribution of patients according to their spirometric classification of GOLD 2015 was as follows: 16 were mild stage, 14 were moderate stage, and 6 were severe stage. 19 patients who underwent a curative resection with normal lung function were recruited in control group. For all enrolled individuals, 19 were patients with lung adenocarcinomas, 22 were patients with squamous lung cancer, and 8 of them were individuals with benign lesions confirmed by histology. No significant differences in age, weight, or height were observed among the subjects (data not shown). Of note, the never smoker group contained an approximately equal number of males and females, but subjects in ex-smoking and current smoking groups were predominantly males. In addition, no difference in cigarette consumption (pack-years) of smoking history was found between non-COPD patients and COPD patients with different degrees of disease severity (*p* = 0.13). Other clinical characteristics and lung function data of the COPD patients and non-COPD patients were presented in Table 1. The selected criteria of the values of pulmonary function, FEV1% pred, FEV1, FVC, and FEV1/FVC%, were significantly different in patients with COPD compared to the patients without COPD (*p* = 0.003 for FVC, and *p* = 0.000 for FEV1% pred, FEV1, and FEV1/FVC%) (Table 1).

### 3.2. An Increased Volume of Airway Smooth Muscle (ASM) Mass in Small Airway during the Progression of COPD

**Figure 1:**
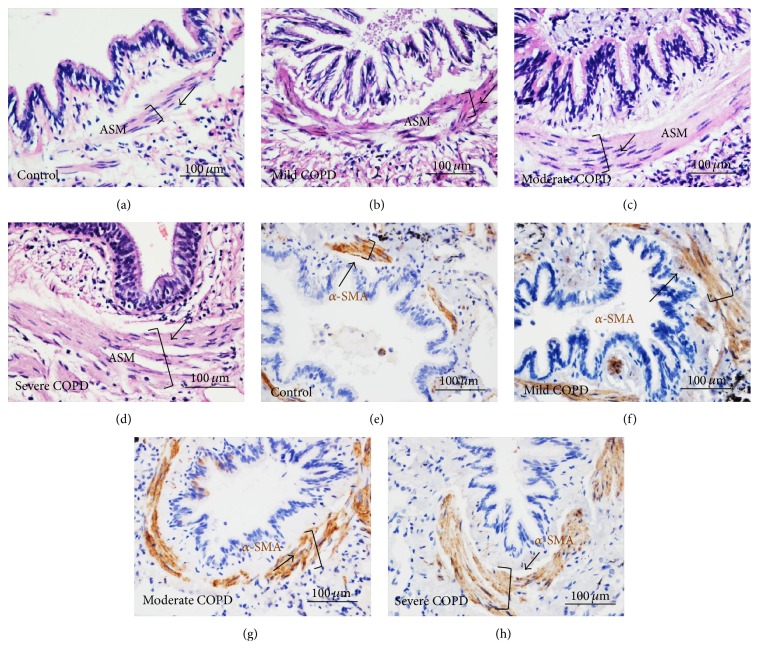
Morphological and immunohistological characterization of airway smooth muscle mass in small airway of COPD lungs. (a)–(d) Morphological characterization of small airways of COPD lung by HE staining; an increased thickness of ASM (brackets and arrows) was observed with disease severities. (a)–(d) Representative images of small airways in control (a), mild (b), moderate (c), and severe (d) COPD lungs. (e)–(h) Immunohistological feature of ASM mass determined by IHC using anti-*α*-SMA antibody; IHC confirmed an increased thickness of AMS (brackets and arrows) with the disease severity. (e)–(h) Representative IHC images of small airways in control (e), mild (f), moderate (g), and severe (h) COPD lungs. Both of HE and IHC staining showed an increased ASM mass with the disease severity. Bars in (a)–(h): 100 *μ*m.

**Figure 2:**
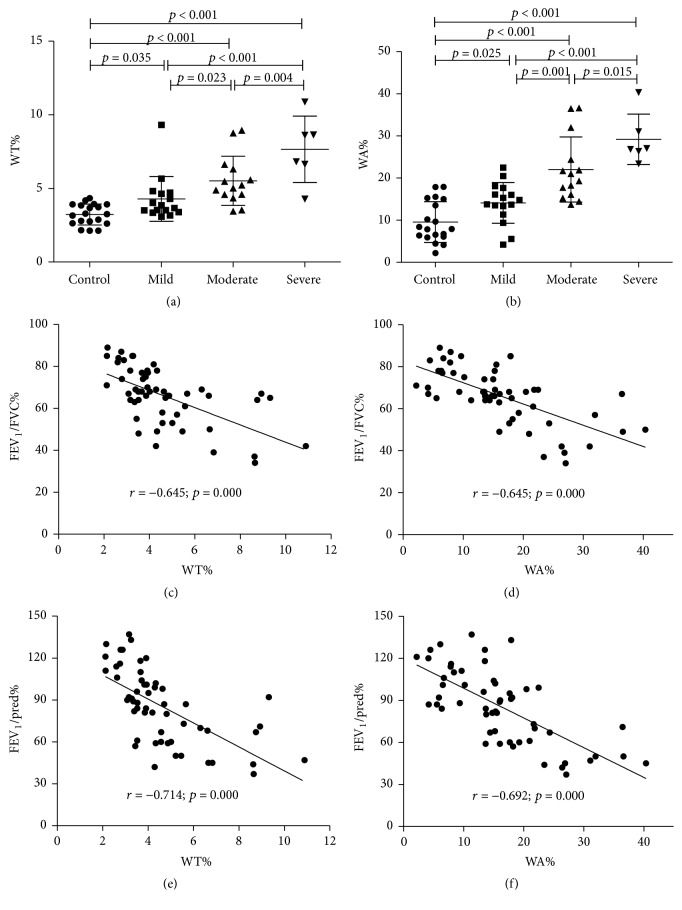
The correlation of the thickness or area of ASM and the progression and pulmonary function in COPD lungs. (a)-(b) The correlation of the COPD disease severity with ASM mass in small airway was evaluated by indexes of the percentages of the thickness (*T*)/external diameter (*D*) of ASM (WT%) and area of ASM/transverse area of small airway (WA%). (a) Values of WT% in small airways from indicated groups with different disease severities. (b) Values of WA% in small airways from indicated groups with different disease severities. (c)–(f) The correlation between the thickness or area of ASM and the pulmonary function in COPD patients. (c) The correlation between WT% and FEV1/FVC% (*r* = −0.645; *p* = 0.000). (d) The correlation between WA% and FEV1/FVC% (*r* = −0.645; *p* = 0.000). (e) The correlation between WT% and FEV1% pred (*r* = −0.714; *p* = 0.000) and (f) the correlation between WA% and FEV1% pred (*r* = −0.692; *p* = 0.000). The data showed a negative association of values of WA% and WT% with pulmonary function. Statistical analysis was performed by one-way ANOVA and *t*-test for comparison between groups using the GraphPad Prism version 5 software. Data was presented as the mean ± SD. Spearman's correlation coefficient and *p* values are as indicated.

ASM mass of small airway in human lung from control subject and patients with COPD was evaluated by morphological analysis by HE staining (Figures 1(a)–1(d)) and IHC using antibody against *α*-SMA (Figures 1(e)–1(h)). The result showed an increased ASM mass with the severity of airway limitation. The morphological analysis by HE staining revealed that ASM layer in COPD lung was thicker than that of control samples (Figure 1). In addition, such an increased ASM mass in small airway was found to correlate with the disease severity of COPD, as determined by an index of the percentage of the thickness (*T*)/external diameter (*D*) of ASM (WT%) (Figure 2(a)) and the percentage of the area of ASM/transverse area of small airway (WA%) (Figure 2(b)). Importantly, the pulmonary functional correlation analysis further demonstrated that the values of above WT% and WA% were inversely associated with pulmonary function, as the values of FEV1% pred and FEV1/FVC% were used as functional indexes (Figures 2(c)–2(f)). The correlation coefficients were *r* = −0.645 (*p* = 0.000) for WT% and FEV1/FVC% (Figure 2(c)), *r* = −0.645 (*p* = 0.000) for WA% and FEV1/FVC (Figure 2(d)), *r* = −0.714 (*p* = 0.000) for WT% and FEV1% (Figure 2(e)), and *r* = −0.692 (*p* = 0.000) for WA% and FEV1% pred (Figure 2(f)).

### 3.3. An Enhanced Proliferative Capacity of ASM Cells and Deposition of Extracellular Matrix in Small Airway of COPD Lungs

**Figure 3:**
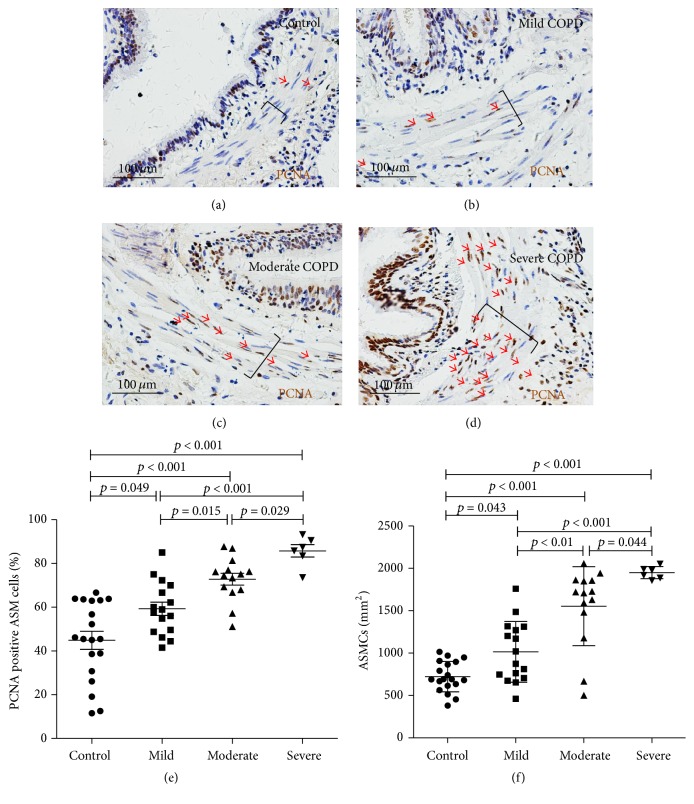
The expression of proliferation of ASM cells in COPD lungs. (a)–(d) The expression of proliferative marker PCNA in ASM cells determined by an IHC assay; an increased number of PCNA positive ASM cells (arrowheads) were observed with disease severities. (a)–(d) Representative IHC images of PCNA for small airway of control (a), mild (b), moderate (c), and severe (d) COPD lungs. (e)-(f) Quantitative analysis of the proliferation of ASM cells and disease severity in COPD. (e) The percentage of PCNA positive ASM cells and (f) the numbers of ASM cells per square millimeter in the area of ASM in small airway were increased with disease severities. Data was presented as the mean ± SD in (e) and (f). Bars in (a)–(d): 100 *μ*m; brackets in (a)–(d) represented the ASM layers.

**Figure 4:**
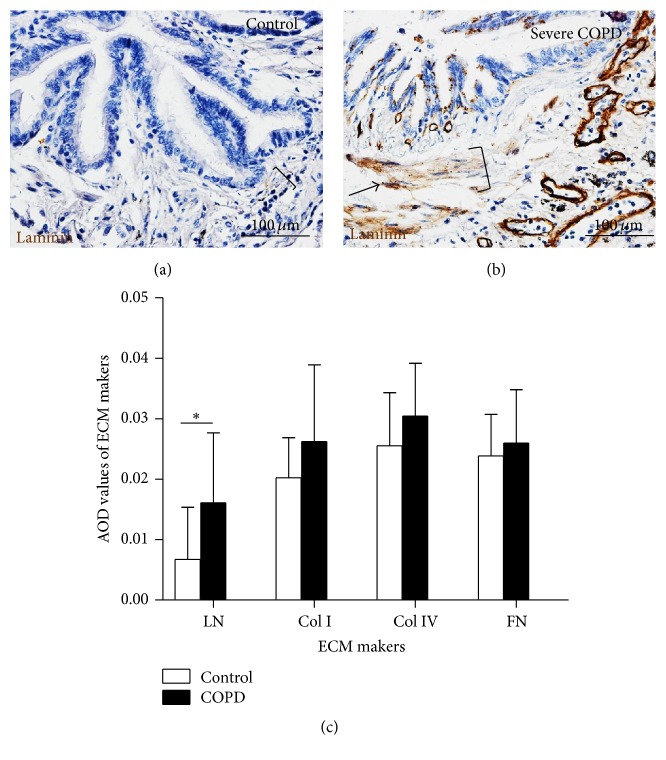
The expression of extracellular matrix markers in ASM of COPD lungs. (a)-(b) The expression of extracellular matrix (ECM) marker laminin in ASM determined by an IHC assay. (a)-(b) Representative IHC images of laminin staining for small airway of control (a) and severe COPD (b) lungs showed a more abundant laminin (arrow) in ASM of lung from severe COPD. (c) The expressions of indicated ECM markers laminin (LN), collagen I (Col I), collagen IV (Col IV), and fibronectin (FN) in ASM of control lungs (*N* = 19) and COPD lungs (*N* = 36) were semiquantified on IHC slides by average optic density (AOD). The data showed a significantly elevated expression of laminin, but not collagen I; collagen IV and fibronectin were observed in ASM of COPD small airway. Data was presented as the mean ± SD in (c). Compared with the control group, ^*∗*^ *p* < 0.05 in (c). Bars in (a) and (b): 100 *μ*m; brackets in (a) and (b) represented the ASM layers.

Interestingly, IHC staining evaluation showed more abundant proliferative cells in ASM and epithelial cells of small airway in COPD lungs relative to the controls, as ascertained by cell proliferative marker PCNA (Figures 3(a)–3(d)). Data from the quantitative analysis further exhibited that the proliferation of ASM cells in small airway was increased with the progression of COPD diseases when it was ascertained by the percentage of PCNA positive ASM cells (Figure 3(e)), and the numbers of ASM cells per square millimeter in the area of ASM in small airway (Figure 3(f)), suggesting that the proliferative capacity of ASM cells was enhanced with the severity of airflow limitation of COPD. In addition, an increasing secretion and deposition of ECM markers is a hallmark of airway remodeling in COPD. The IHC staining result also revealed more deposition of laminin, a marker of ECM in the ASM cells and epithelial cells of small airway in severe COPD lungs relative to controls (Figures 4(a) and 4(b)). Semiquantitative analysis further demonstrated there was a significant difference in the deposition of laminin molecules between the COPD and control lungs. However, no significant difference was observed in the abundance of other ECM markers including collagen I (Col I), collagen IV (Col IV), and fibronectin (FN) (Figure 4(c)).

### 3.4. An Elevated Expression of NOX4 in ASM of Small Airway in COPD Lungs

**Figure 5:**
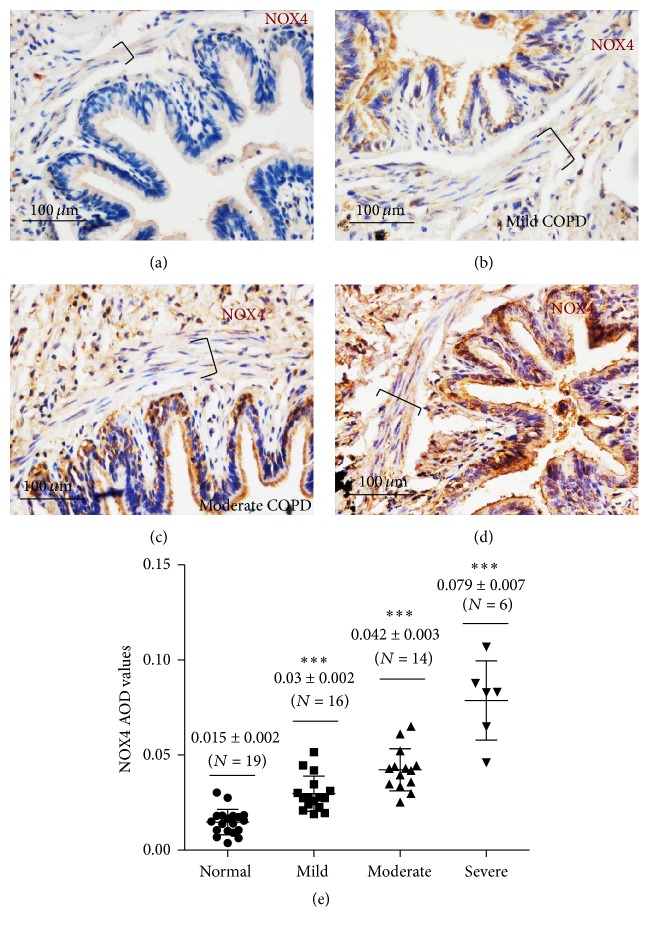
An elevated expression of NOX4 in ASM cells of small airway in COPD lung. (a)–(d) The abundance of NOX4 protein in lung tissues determined by an IHC assay. (a)–(d) The expression of NOX4 protein in the small airway of human lung from control subject (a), and patients with mild (b), moderate (c), and severe (d) COPD were determined by IHC with antibody against NOX4. An increased abundance of NOX4 protein was observed in ASM layer of small airway (bracket) with the disease severity of COPD. (e) The levels of NOX4 protein in ASM of indicated groups were semiquantitatively evaluated by accessing average optical density (AOD) using the IPP6.0 software, which correlated to the disease severity of COPD. Statistical analysis was performed by one-way ANOVA and *t*-test for comparison between groups using the GraphPad Prism version 5 software. Data was presented as the mean ± SD, which were indicated in (e). Bars in (a) and (b): 100 *μ*m; brackets in (a) and (b) represented the ASM layers. ^*∗∗∗*^ *p* < 0.0001.

**Figure 6:**
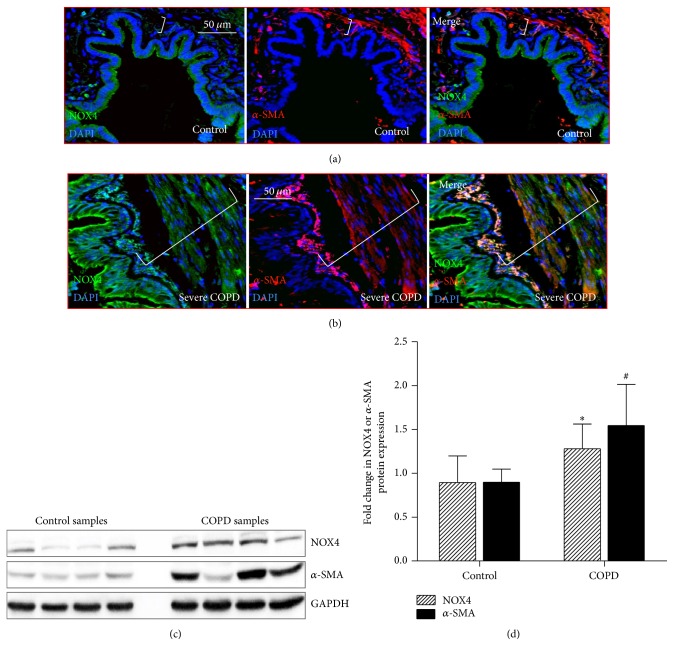
Colocalized NOX4 and *α*-SMA proteins in ASM cells of small airway in COPD lung. (a)-(b) Colocalization of NOX4 expression and *α*-SMA in ASM cells of small airway by immunofluorescent assay. (a), (b) Representative images of colocalized expressions of NOX4 (green) and *α*-SMA (red) proteins in the small airway of human lung from a control subject (a) and a patient with severe COPD (b). (c) Immunoblotting analysis determined the abundance of NOX4 and *α*-SMA proteins in lung tissues of control subjects and COPD patients. (d) Semiquantitative analysis of the abundance of proteins of immunoblots in (c) by a densitometric analysis. More abundant NOX4 was found in COPD tissues. GAPDH was used as internal control for loading of samples, and 6-diamidino-2-phenylindole (DAPI, blue) was used for nuclear staining in (a) and (b). Bars in (a) and (b): 50 *μ*m; brackets in (a) and (b) represented the ASM layers. Compared to controls, ^*∗*^ *p* < 0.05 for NOX4.

NOX4 has been recognized to be involved in the proliferation of vascular smooth muscle and fibroblast in pulmonary fibrosis and asthma [14, 19]. In order to explore whether NOX4 is involved in ASM of small airway during the cause of COPD pathogenesis, the expressions of NOX4 in the small airway of human lung from control subject and patients with COPD were determined by IHC. Interestingly, more abundant NOX4 protein was detected in ASM cells of COPD small airways relative to control airways (Figures 5(a)–5(d)). Of interest, an elevated NOX4 expression was enhanced along with the disease severity as evaluated by an IHC staining (Figures 5(a)–5(d)) and a semiquantitative assay of the staining for COPD lung tissues with different severities of disease (Figure 5(e)). Importantly, an immunofluorescent (IF) analysis further uncovered that the NOX4 and *α*-SMA proteins were colocalized in ASM cells of small airway as determined by a dual IF staining; more abundant NOX4 and *α*-SMA proteins were observed in COPD ASM cells than that in control lungs (Figures 6(a) and 6(b)). In addition, an increased expression of NOX4 was found to accompany an upregulated *α*-SMA expression in COPD lungs (Figures 6(a) and 6(b)). Immunoblotting analysis further confirmed more abundant NOX4 and *α*-SMA proteins in the small airway specimens from COPD patients as compared with that from control subjects (Figure 6(c)).

### 3.5. A Correlation of NOX4 Expression and ASM Mass and Lung Function

**Figure 7:**
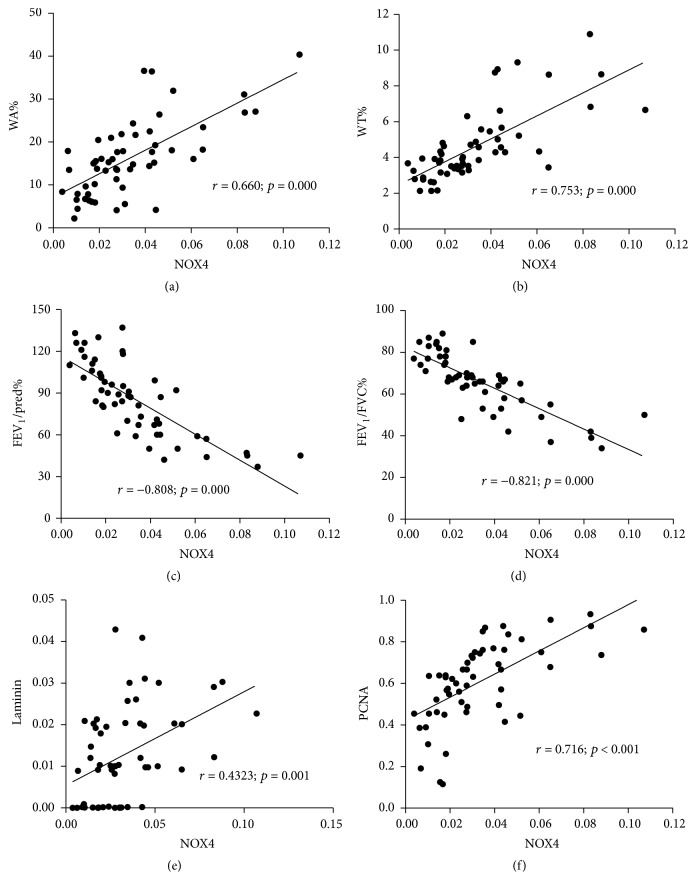
Correlation of the expression of NOX4 and ASM mass, pulmonary function, or ASM cell proliferation in COPD lungs. (a) Correlation between the expression of NOX4 and the WA% in small airway (*r* = 0.660; *p* = 0.000). (b) Correlation between the expression of NOX4 and WT% in small airway (*r* = 0.753; *p* = 0.000). (c) Correlation between the expression of NOX4 and pulmonary function index FEV1% pred (*r* = −0.808; *p* = 0.000). (d) Correlation between the expression of NOX4 and pulmonary function index FEV1/FVC% (*r* = −0.821; *p* = 0.000). (e) Correlation between the expression of laminin and NOX4 in ASM of small airway in indicated groups as determined by AOD values (*r* = 0.432; *p* = 0.001). (f) Correlation between expression of NOX4 and PCNA as determined by AOD values (*r* = 0.716; *p* = 0.001). The data showed a positive association of NOX4 level with ASM mass and proliferative capacity and a negative correlation with the pulmonary function. Spearman's correlation coefficient and *p* values are as indicated.

We next sought to know whether the evoked expression of NOX4 was correlated with small airway ASM mass and pulmonary function in COPD. Correlation analysis demonstrated that the level of NOX4 protein in ASM of small airway was positively correlated with ASM mass, ECM deposition, and ASM cell proliferation but inversely associated with pulmonary function. The correlation coefficients were *r* = 0.660 (*p* = 0.000) for NOX4 and WA% (Figure 7(a)), *r* = 0.753 (*p* = 0.000) for NOX4 and WT% (Figure 7(b)), *r* = −0.808 (*p* = 0.000) for NOX4 and pulmonary function indexes FEV1% pred (Figure 7(c)), *r* = −0.821 (*p* = 0.000) for NOX4 and FEV1/FVC% (Figure 7(d)), *r* = 0.432 (*p* = 0.001) for NOX4 and ECM marker laminin (Figure 7(e)), and *r* = 0.716 (*p* = 0.001) for NOX4 and proliferative marker PCNA expression (Figure 7(f)). These data clearly suggest that the NOX4 may play an important role in the ASM remodeling and progression of COPD lungs.

### 3.6. A Correlation of NOX4 and TGF-*β* Proteins in ASM of Small Airway in COPD Lung

**Figure 8:**
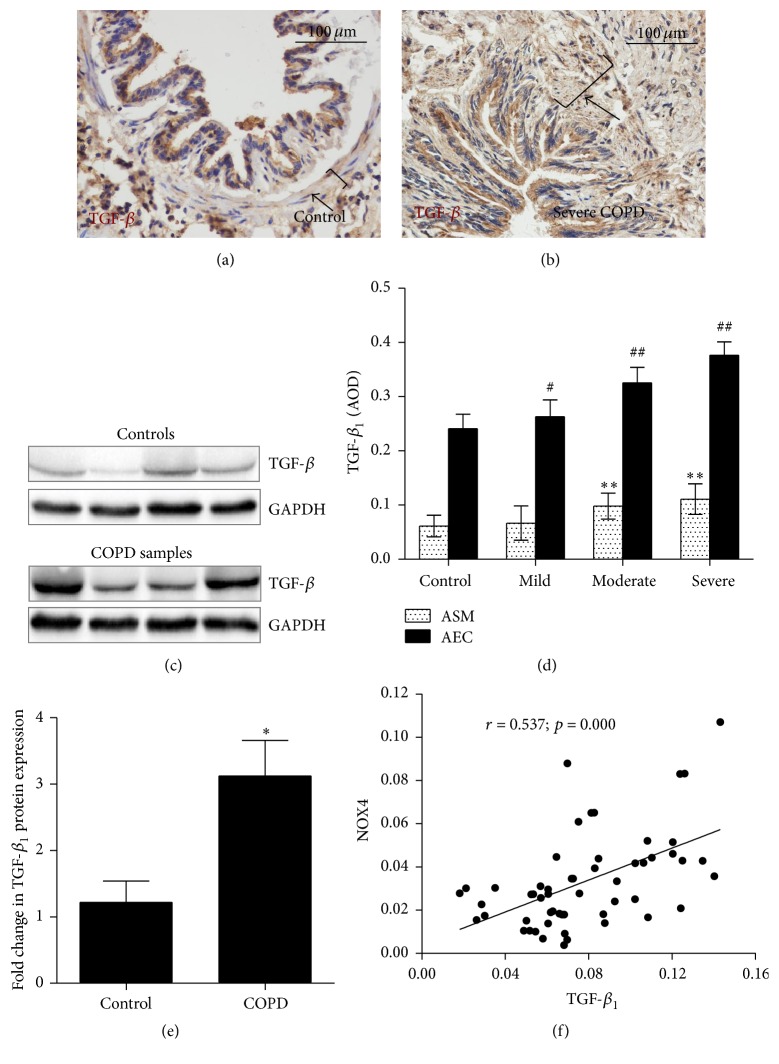
Correlation of the expression of NOX4 and TGF-*β* in ASM of small airway. (a), (b) The expression of TGF-*β* was determined by IHC with antibody against TGF-*β*. (a), (b) Representative images of IHC for TGF-*β* expression in small airways of control lung (a) and severe COPD lung (b). (c) Immunoblotting analysis determined the abundance of TGF-*β* protein in small airways of control subjects (a, c, e) and COPD patients (b, d, f). More abundant TGF-*β* was observed in COPD airways. (e) Comparison of the expression of TGF-*β* in ASM and airway epithelial cells (AEC) of control lungs (*N* = 19) and mild COPD (*N* = 16), moderate COPD (*N* = 14), and severe COPD (*N* = 6) lungs as determined by AOD values. (e) Semiquantitative analysis of the abundance of proteins of immunoblots in (c) by a densitometric analysis; the result showed the fold changes of TGF-*β* in COPD lungs over non-COPD lungs. (f) Correlation between the expression of NOX4 and TGF-*β* in ASM of small airway (*r* = 0.537; *p* = 0.000). GAPDH was used as internal control for loading of samples in (c). Statistical analysis was performed by one-way ANOVA and *t*-test for comparison between groups using the GraphPad Prism version 5 software. Data was presented as the mean ± SD. Spearman's correlation coefficient and *p* values are as indicated. Compared to the controls, ^*∗∗*^ *p* < 0.01 in ASM; ^#^ *p* < 0.05 and ^##^ *p* < 0.01 in AEC. Bars in (a) and (b): 100 *μ*m; brackets in (a) and (b) represented the ASM layers. Compared to controls, ^#^ *p* < 0.05 for *α*-SMA.

Since TGF-*β* signaling plays a pivotal role in airway modeling of chronic pulmonary diseases including COPD, pulmonary fibrosis, and asthma, which also interacted with NOX4 signaling [17, 37], the expression of TGF-*β* in ASM of small airway was determined by IHC and immunoblotting assays. IHC results showed an abundant TGF-*β* expressed in airway epithelial cells (AECs) and interstitial cells including ASM cells of both control and COPD lungs, despite the fact that the predominant TGF-*β* was observed in AECs (Figures 8(a) and 8(b)). Immunoblotting assay further revealed an overall more abundant TGF-*β* protein in COPD lungs as compared with the controls, although variations between donor tissues were observed (Figures 8(c) and 8(e)). The result of a semiquantitative analysis of the IHC staining assay showed that the expression of TGF-*β* in both of ASM cells and AECs in COPD lungs was increased along with the disease severity in comparison with the controls (Figure 8(d)). Importantly, correlation analysis further demonstrated that the level of TGF-*β* protein in ASM of small airway was correlated with the expression of NOX4, with a coefficient *r* = 0.537 (*p* = 0.000) (Figure 8(f)).

## 4. Discussion

Airway remodeling is an important cause of airway narrowing and airflow limitation in chronic pulmonary diseases such as COPD and asthma, which is mainly attributed to alterations of composition and structure of airway epithelium, submucosal glands, blood vessels, extracellular matrix deposition, and airway smooth muscle [24, 28, 30]. An increasing number of studies have demonstrated that airway smooth muscle (ASM) remodeling is strongly correlated with the severity and progression of chronic pulmonary disorders with airway obstruction, including COPD and asthma, in which ASM is the key end-effector of bronchospasm and acute airway narrowing [24, 38, 39]. However, owing to the difficulty encountered during isolation of these long thin cells from the dense extracellular matrix of small airway with a diameter less than 2 mm, and the availability of human lung tissues, our understanding of the role and mechanism of ASM in small airway remodeling during the pathogenesis of COPD is limited. Equally noteworthy, NOX protein derived ROS have been implicated in regulation of cytoskeletal remodeling and now have been recognized to play essential roles in both of the physiology and pathology in lung [40, 41]. In this regard, the role and underlying mechanism of NOX4 in the vascular and airway remodeling, fibroblast proliferation, and vascular smooth muscle hyperplasia/hypertrophy during the progression of asthma, and particularly the IPF, have been well documented [14–16]. Importantly, an evoked expression of NOX4 in ASM has recently been demonstrated to mediate intrinsic ASM hypercontractility in asthmatic lung [27]. However, the expression and functional consequence of NOX4 in ASM of small airway during the development and pathogenesis in COPD remain elusive.

In the present report, we interrogated the expression of NOX4 protein in ASM of small airway of patients with COPD. Our results uncovered an increased ASM mass in small airway of COPD lung relative to the control lungs. Such a hyperreactivity or remodeling of ASM was enhanced with cell proliferative capacity (hyperplasia) and disease severity, and it was inversely correlated with the pulmonary function in COPD patients. This finding was consistent with results reported in a previous study in patients with COPD [42]. However, we could not rule out whether the ASM hypertrophy seen in asthmatic lung was involved in the ASM remodeling found in small airway of COPD in this study. ASM remodeling in asthmatic lung was suggested to be a consequence of ASM hypertrophy/hyperplasia and ECM deposition [24, 30, 38]. Importantly, we found that NOX4 was elevated in ASM of COPD small airway along with the severity of disease. Correlation analysis further demonstrated that the level of NOX4 in ASM was inversely associated with the pulmonary function but positively correlated with the abundance of ECM marker laminin and TGF-*β* in small airway of COPD lungs. These data imply that NOX4 is a main regulator in oxidative stress during small airway remodeling, and the regulatory role of NOX4 may be at least in part through a mechanism involving TGF-*β* signaling, which merits further investigation.

ECM deposition is another hallmark of airway remodeling. In this context, both of AECs and ASM have shown a capacity of ECM secretion that leads the deposition of ECM, which has a strong impact on the proliferation, adhesion, and contractility of ASM [19, 24, 25, 43]. Of note, components of ECM during airway remodeling may be varied among diseases, and the predominant ECM proteins secreted by ASM in COPD include fibronectin, laminin, collagen I, and collagen IV [30, 44]. In line with these findings, increased deposition of laminin was observed in the ASM and small airway of COPD lung in this study, despite the fact that the expression of other examined ECM markers, fibronectin, collagen I, and collagen IV, was not significantly changed in small airways between the lung without hyperreactivity disease and COPD. This result was not in agreement with a finding reported in a previous study by Parameswaran et al., in which the authors stated an elevated expression of all four ECM proteins, including the fibronectin in small airway of COPD [44]. We reasoned that such a discrepancy of fibronectin expression reported in different studies might be caused by different criteria employed for clinical sample collection. Nevertheless, our results clearly indicated that an evoked secretion of ECM occurred in ASM of small airway during the pathogenesis of COPD, and the deposition of ECM in turn promoted the progression of airway remodeling and disease activity.

It has been recently recognized that an imbalance of oxidant/antioxidant in the lung contributes to the development and progression of COPD [6]. Indeed, the degree of oxidative tissue or systematic damage in COPD patients is more often and/or severe relative to healthy individuals, which is thought to be strongly correlated with the physiopathological changes and disease manifestation (inflammation, emphysema, airway remodeling, and mucus hypersecretion) in COPD [2, 6]. In this context, NOX-produced ROS plays a significant role in the pathogenesis of COPD, including the airway remodeling, such ASM contraction in small airways [42]. In addition, the NOX4 was found to be a main ROS producer that is constructively expressed in the vascular smooth muscle and ASM in lung of asthmatic patients and COPD patients, implying that it might be a pivotal regulator of abnormal smooth muscle function in these diseases [19, 27].

In smooth muscle cells, inflammatory cytokines, hypoxia, and TGF-*β* have been identified as NOX4 inducers, among which the TGF-*β* signaling is a major player which received most attention, owing to its fundamental functions in the course and pathogenesis of pulmonary fibrosis and obstructive lung diseases [45]. In terms of ASM, Michaeloudes et al. found that TGF-*β* could trigger intracellular ROS release in ASM cells by upregulation of NOX4 but downregulation of MnSOD and catalase, accompanied by elevated ROS and IL-6 production [46]. Mechanistically, the TGF-*β* can bind its receptor ALK5/T*β*RII and activate phosphorylation of Smad2/3 and PI3K and accordingly activate the transcription of NOX4 and upregulated NOX4 expression, which sequentially increased the intracellular ROS level [37]. The increased ROS can in turn activate TGF-*β*/Smad signaling by promoting conversion of latent TGF-*β* to an active form, enhancing the phosphorylation of ALK5 and Smad2/3, as well as the activation of JNK and p38 [37]. Consequentially, the activated TGF-*β*/ROS signaling promotes ASM remodeling by facilitating muscle cell proliferation and fibroblast myofibrosis and enhanced ECM synthesis and deposition [37, 47]. Consistent with this notion, our results revealed that the expression of NOX4 and TGF-*β* was elevated in the epithelial cells and ASM cells of small airways of COPD lung, and the NOX4 level was tightly correlated with the TGF-*β* expression in ASM. These data suggest a mechanism by which the interaction between NOX4 and TGF-*β* signaling may play a critical role in the small airway remodeling in general, and ASM remodeling in particular, during the pathogenesis and course of COPD.

Of interest, we also noted that a more evaluated expression of NOX4 and TGF-*β* relative to that of controls was also observed in epithelial cells of small airway in COPD lungs. Indeed, many pulmonary cell types, including endothelial cells, neutrophils, alveolar macrophages, and alveolar epithelial cells, are major sites of ROS generation [48], in which ROS have long been recognized to play key roles in the pathogenesis of several chronic pulmonary diseases, such as asthma, pulmonary fibrosis, and COPD [7]. A high level of TGF-*β* was previously reported in AECs of COPD lungs [49], which has an important implication in the dedifferentiation [50], epithelial-to-mesenchymal transition [51], and permeability [52] of airway epithelia in COPD. In addition, an upregulated NOX4 level in AEC was also recently found in influenza A virus infected lung epithelial cells, in which NOX4 mediated the major ROS production in inflammatory cells [53].

Equally noteworthy, smoking has been suggested to be one of the main causes of COPD. Cigarette smoking can exacerbate oxidative burden involved in airway damage and remodeling; however the involvement of NOX4 signaling in the smoking-induced pathogenesis of the airway injury has not been investigated. In order to evaluate whether smoking has an impact on the expression of NOX4 and TGF-*β*, as well as ASM remodeling, parameters including SMA-WT% and SMA-WA% (as indexes of ASM mass for ASM remodeling), and the expression of NOX4 and TGF-*β* between the control non-COPD group, current smokers with COPD, ex-smoker with COPD, and never smokers with COPD. Unexpectedly, no significant difference of above examined parameters between these groups was observed, except the non-COPD controls showing better pulmonary functions, less abundant ASM mass, and NOX4 and TGF-*β* proteins, as compared with all COPD groups (Table 2). Notably, our result could not rule out whether the smoking has an impact on the alteration of NOX4 expression and ASM remodeling in the pathogenesis OF COPD, since the sample size was too small to draw a conclusion in this study.

Together with our findings in this report, these studies suggested that NOX4 could induce the generation of ROS, which subsequently promoted a morphological change and dysfunction of ASM and accordingly led the remodeling of airways in COPD lungs. However, mechanisms underpinning the NOX4-induced ASM hypertrophy/hyperplasia remain elusive, which need further investigation. Nonetheless, our study also implies that the NOX4 signaling may be a novel target for investigating mechanisms of airway remodeling and developing agents for COPD treatment. However, the clinical significance of an elevated NOX4 in AEC of COPD lung currently remains elusive and is worthy of further investigation.

## 5. Conclusion

In summary, in the present study, we interrogated the expression of NOX4 and TGF-*β* in the ASM of small airways in patients with COPD by an immunohistochemistry assay. The correlation between levels of NOX4 and TGF-*β* in ASM and pulmonary functions was also analyzed. The results revealed an increased expression of NOX4 and TGF-*β* with the severity of airflow limitation in ASM of small airway of COPD lung, which was inversely associated with the pulmonary function. Our data thus suggest that NOX4 may be a key player in the ASM remodeling during the course and pathogenesis of COPD, and the mechanism underpinning its biological function in the pathogenesis of COPD and other obstructive pulmonary disorders merits further investigation.

## Figures and Tables

**Table 1:** Demographics of patients with COPD and non-COPD control subjects; data was presented as mean ± SD.

Demographics	Non-COPD cohorts	Patients with COPD			*F*	*p*
		Mild	Moderate	Severe		
Subjects	19	16	14	6	NS	NS
Age (s)	56.53 ± 7.33	58.88 ± 9.02	58.36 ± 8.67	55.83 ± 11.55	0.33	0.803

Gender						
Male	10	12	9	6	NS	NS
Female	9	4	5	0	NS	NS

Smoking status and cigarette consumption						
Never smoking	11	6	5	1	NS	NS
Ex-smoking	1	3	3	3	NS	NS
Current smoking	7	7	6	2	NS	NS
Pack-years	11.05 ± 14.49	18.63 ± 17.59	21.43 ± 18.34	28.33 ± 20.41	1.97	0.130

Pulmonary functions						
FEV1% pred	108.89 ± 15.22	93.94 ± 14.74	62.29 ± 7.30	43.33 ± 3.50	61.81	0.000
FEV1	2.90 ± 0.57	2.55 ± 0.57	1.72 ± 0.26	1.41 ± 0.37	23.93	0.000
FVC	3.65 ± 0.69	3.83 ± 0.69	2.98 ± 0.42	3.41 ± 0.62	5.19	0.003
FEV1/FVC%	79.63 ± 5.48	66.56 ± 1.90	58.21 ± 7.32	40.67 ± 5.50	97.07	0.000

Data was presented as mean ± SD. NS: no statistical difference; FEV1: forced expiratory volume in one second; FVC: forced vital capacity. *F* value is the homogeneity of variance between two groups. *p* values were given as COPD versus non-COPD groups.

**Table 2:** Comparisons of ASM mass and the abundance of NOX4 and TGF-*β* proteins between smokers and nonsmokers (mean ± SD).

		*n*	SMA-WT%	SMA-WA%	NOX4 (A.U)	Epithelial TGF-*β* (AU)	ASM TGF-*β* (AU)
Non-COPD^a^	Never smokers	11	3.33 ± 0.77	10.22 ± 5.47	0.014 ± 0.007	0.232 ± 0.024	0.059 ± 0.023
	Smokers	8	3.08 ± 0.65	8.65 ± 4.00	0.015 ± 0.006	0.253 ± 0.027	0.0627 ± 0.019
	*F*		0.129	1.956	0.097	0.034	0.028
	*t*		−0.731	−0.687	0.314	1.817	−0.343
	*p*		0.475	0.502	0.757	0.087	0.736

COPD^b^	Never smokers	12	5.39 ± 1.99	20.05 ± 7.81	0.037 ± 0.018	0.294 ± 0.041	0.085 ± 0.040
	Current smokers	15	5.10 ± 1.82	18.81 ± 7.95	0.048 ± 0.024	0.304 ± 0.014	0.092 ± 0.028
	Ex-smokers	9	5.61 ± 2.61	20.73 ± 10.29	0.042 ± 0.020	0.327 ± 0.058	0.078 ± 0.034
	*F*		0.176	0.158	0.79	1.07	0.464
	*p*		0.839	0.854	0.462	0.354	0.633

COPD^c^	Never smokers	12	5.39 ± 1.99	20.05 ± 7.81	0.037 ± 0.018	0.294 ± 0.041	0.085 ± 0.040
	Smokers	24	5.29 ± 2.11	19.53 ± 8.73	0.045 ± 0.022	0.312 ± 0.056	0.087 ± 0.030
	*F*		0.003	0.051	0.737	3.308	1.101
	*t*		−0.132	−0.173	1.091	0.993	0.099
	*p*		0.896	0.863	0.283	0.328	0.992

Data was presented as mean ± SD. *F* value is the homogeneity of variance between two groups. *p* values were given as never smoker versus smoker groups. ^a^Since there was only one ex-smoker in non-COPD group, the ex-smoker and current smokers were included in one smoker group; ^b^COPD patients were grouped in never smokers, ex-smokers, and current smokers for analysis; ^c^COPD subjects were grouped in never smokers and smokers (ex-smoker and current smoker) for analysis.

## Abbreviations

AOD: Average optical density
ASM: Airway smooth muscle
COPD: Chronic obstructive pulmonary disease
CSE: Cigarette smoke extract
DUOX: Dual oxidase
ECM: Extracellular matrix
FN: Fibronectin
FEV1: Forced expiratory volume in one second
FVC: Forced vital capacity
GAPDH: Glyceraldehyde-3-phosphatase-dehydrogenase
GOLD: Global initiative for Obstructive Lung Disease
IL: Interleukin
LN: Laminin
LVRS: Lung volume reduction surgery
NADPH: Nicotinamide adenine dinucleotide phosphate
NOX4: Nicotinamide adenine dinucleotide phosphate oxidase 4
PCNA: Proliferating cell nuclear antigen
ROS: Reactive oxygen species
*α*-SMA: Alpha-smooth muscle actin
TGF-*β*: Transforming growth factor *β*
WA%: Percentage of small airway smooth muscle area/bronchial cross-sectional area
WT%: Percentage of small airway smooth muscle thickness/airway diameter.
